# An Unexpected Complication: Obstructive Shock Secondary to Venous Air Embolism

**DOI:** 10.7759/cureus.27922

**Published:** 2022-08-12

**Authors:** Francisco Dá Mesquita Faustino, Ana Sofia Costa, Isabel Ferreira, João João Mendes, Paulo Freitas

**Affiliations:** 1 Critical Care Medicine, Hospital Prof. Doutor Fernando da Fonseca, Lisboa, PRT; 2 Radiology, Hospital Prof. Doutor Fernando da Fonseca, Lisboa, PRT

**Keywords:** id critical care, iv contrast, computerized tomography, obstructive shock, gas embolism

## Abstract

Air embolism is a rare but possibly life-threatening situation. Gas embolism can be arterial, occurring as a complication of a lung biopsy, arterial catheterization, or extracorporeal circulation in the context of cardiopulmonary bypass, or venous, as in cases of venous catheter manipulation (especially with a central venous catheter in a spontaneously breathing patient), pressurized venous infusions, or in a neurosurgical context. Various clinical manifestations are described in the literature, ranging from asymptomatic cases to obstructive shock. Clinical manifestations may include chest pain, dyspnea, nausea and vomiting, altered consciousness, focal neurological deficits, seizures, vertigo, and amaurosis. Physical examination findings may include hypotension and “mill wheel murmur” on chest auscultation. Early diagnosis and treatment are essential to improve the outcome of these patients. Approach and management include placing the patient in the left lateral decubitus and/or Trendelenburg position and on high-flow oxygen. Hyperbaric oxygen therapy is the definitive treatment for arterial gas embolism, which may reduce air emboli size, improve tissue oxygenation, and reduce ischemic lesion. Here, we report the case of a 62-year-old female patient with obesity, hypertension, dyslipidemia, and recovering from coronavirus disease 2019 (COVID-19) with obstructive shock due to venous gas embolism.

## Introduction

Venous gas embolism develops when air or carbon dioxide is introduced intravenously through a pressure gradient (between the entry site and the right atrium). A significant percentage of venous gas embolism is iatrogenic, but some cases also develop due to some form of trauma [1]. Venous gas embolism can occur actively, e.g., through pressurized contrast infusion, or passively, such as through insertion or removal of a central venous catheter (CVC) [2]. Currently, gas embolism has been more recognized with the frequent use of computed tomography (CT) as a diagnostic modality. In most cases, a gas embolus is incidentally detected because small volumes of air within the venous system may not manifest clinically, unlike in cases of arterial air embolism. Although there is no clinical implication in most cases, physicians need to be aware of this phenomenon as large gas emboli in the venous system can have catastrophic hemodynamic consequences [3].

## Case presentation

A 62-year-old female patient with obesity, hypertension, dyslipidemia, and recovering from coronavirus disease 2019 (COVID-19) in an out-of-hospital facility was admitted to our hospital due to acute lithiasic cholecystitis. She underwent videolaparoscopic cholecystectomy complicated postoperatively by hemoperitoneum requiring ligation of the bleeding vessel (an artery branch of the right liver). After seven days because of worsening inflammatory parameters without clear explanation, a contrast CT scan was performed, which excluded abdominal complications. Shortly after the exam, she began to experience acute respiratory failure, requiring a high-concentration face mask with oxygen at 15L/min, with evolution to circulatory shock.

**Figure 1:**
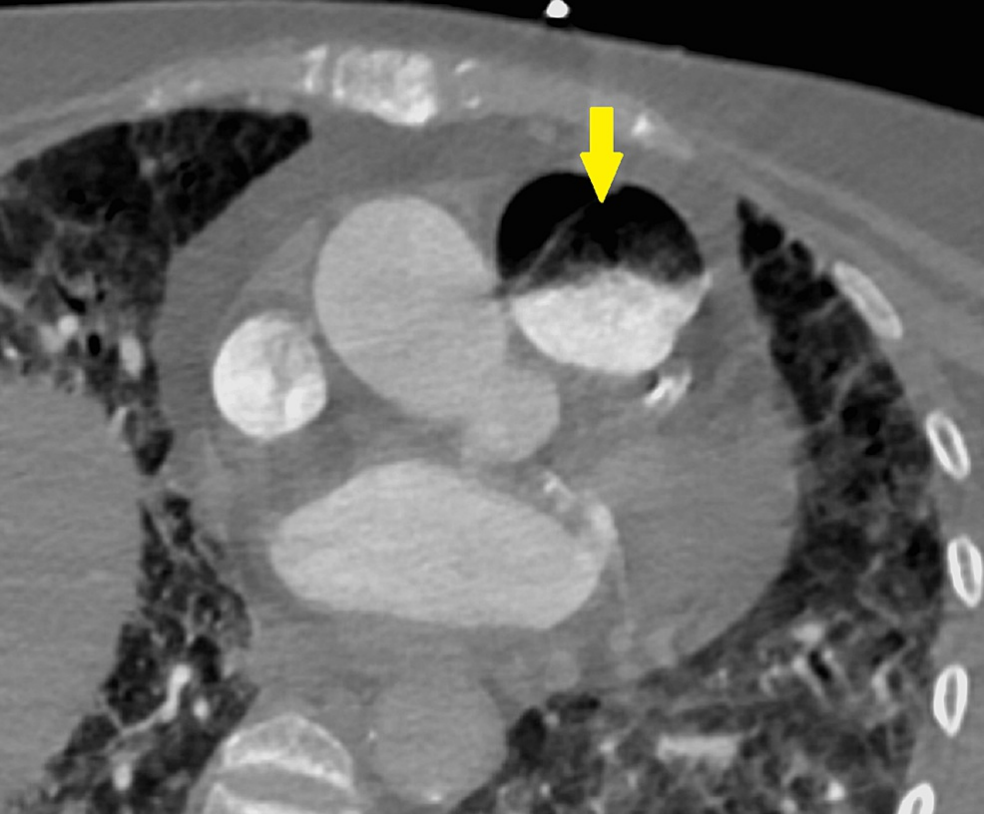
Axial chest computed tomography angiography image at the level of the pulmonary trunk, identifying the air-fluid level in the lumen of the pulmonary trunk (arrow), compatible with gas embolism.

**Figure 2:**
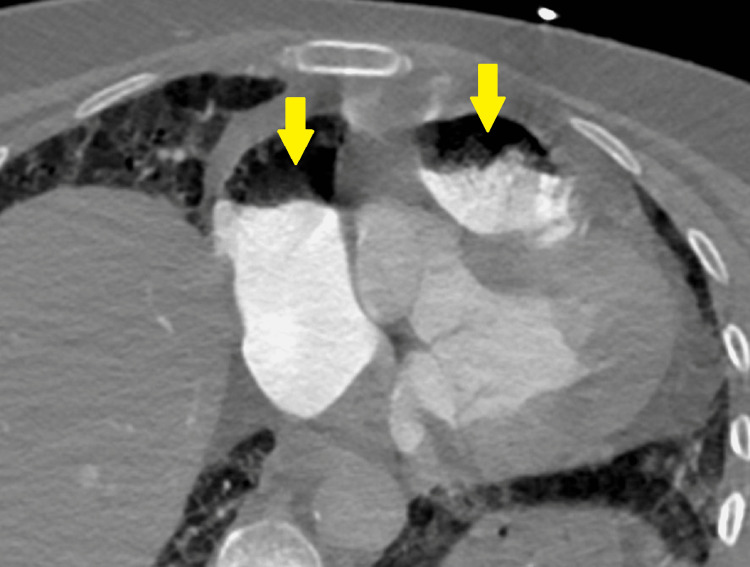
Thoracic computed tomography angiography image in the axial plane at the level of the right cardiac structures, identifying the air-fluid level in the lumen of the right atrium and the right ventricle-pulmonary trunk transition (arrows), compatible with gas embolism.

**Figure 3:**
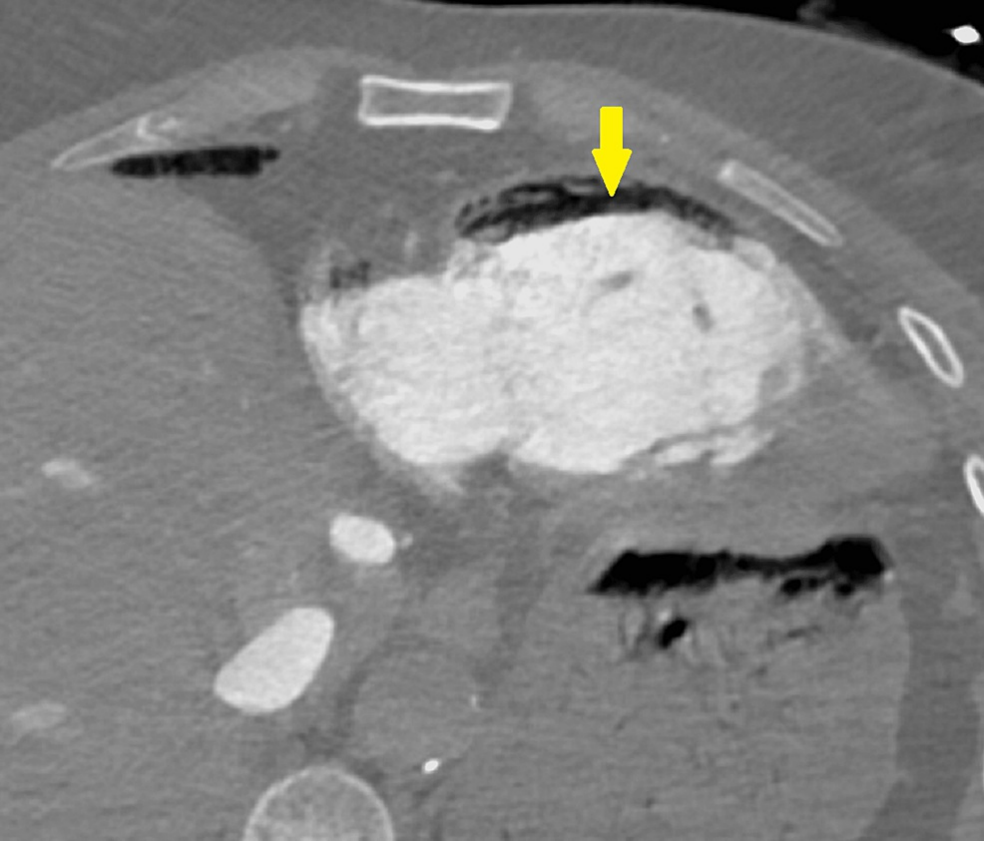
Thoracic computed tomography angiography image in the axial plane at the level of the right ventricle, identifying the air-fluid level in the right ventricular lumen (arrow), compatible with gas embolism.

**Figure 4:**
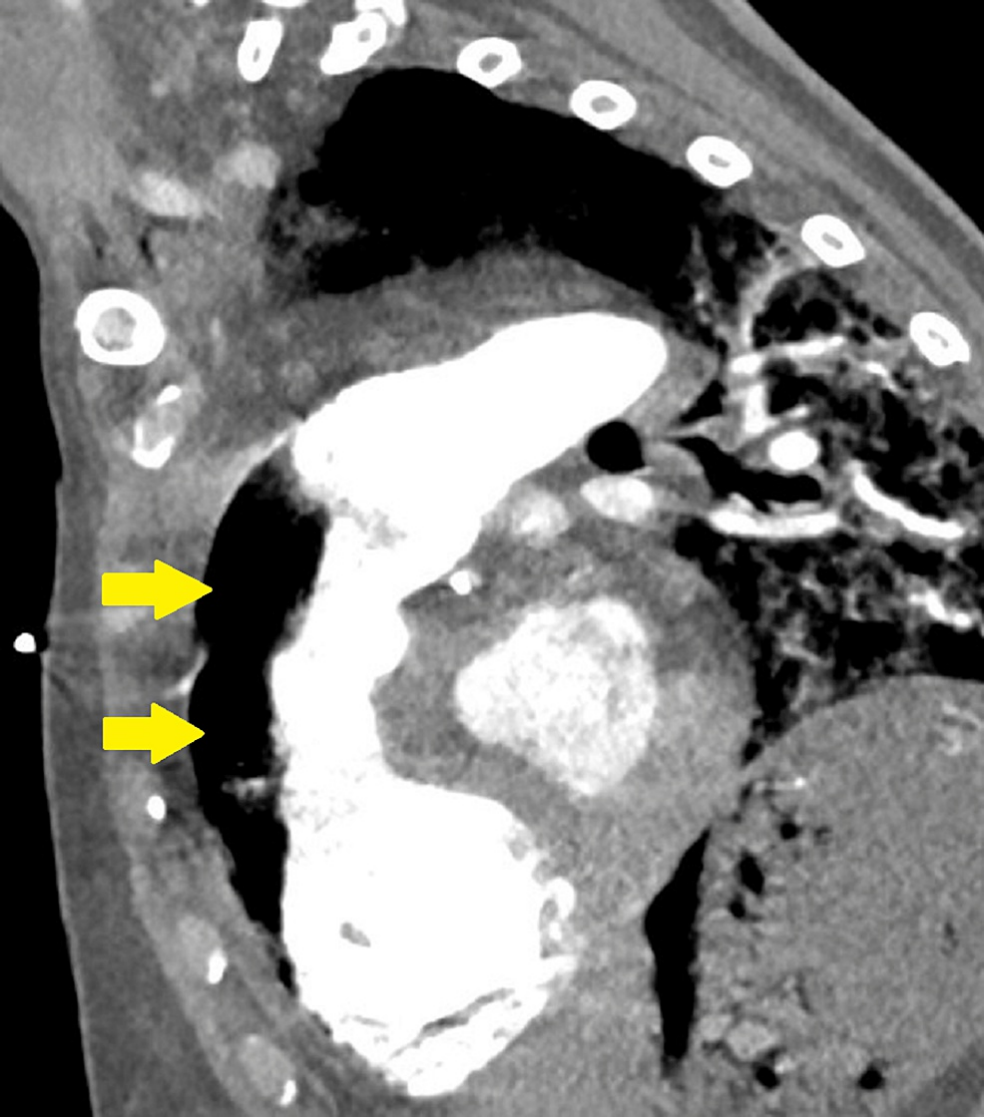
Thoracic computed tomography angiography image in the sagittal plane, identifying the air-fluid level in the lumen of the right ventricle and pulmonary trunk (arrows).

The Rapid Response Team was called, and during stabilization and evaluation of the patient, a transthoracic echocardiogram showed an extreme dilatation of the right heart chambers due to pressure overload associated with autocontrast. An emergent CT pulmonary angiogram was performed (Figures 1-4), which revealed gas embolism with a significant volume of gas in the pulmonary artery trunk, the right heart chambers, and both subclavian and internal jugular veins. During the procedure, the patient underwent cardiorespiratory arrest which resulted from pulseless electrical activity.

Advanced cardiorespiratory support was immediately initiated, and the patient was admitted to the ICU. Approximately 20 mL of air was aspirated through the right jugular CVC, and continuous chest compressions were performed with the patient placed in the Trendelenburg position. Endotracheal intubation and mechanical ventilation were started. An arterial line was placed in the right femoral artery, and a Swan-Ganz catheter was placed in the pulmonary artery (flow-guided placement), which additionally aspirated approximately 10 mL of air. After 15 minutes, she returned to spontaneous circulation still requiring vasopressor support with norepinephrine and volume replacement with crystalloids, colloids, and blood components.

**Figure 5:**
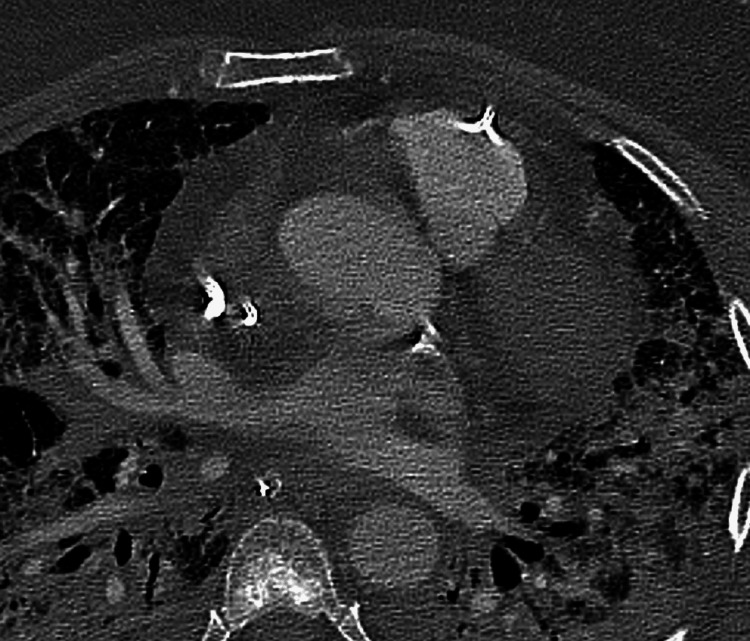
Thoracic computed tomography angiography image in the axial plane, without signs compatible with gas embolism in the lumen of the right ventricle and pulmonary trunk.

The patient's condition improved, with resolution of shock in two days and weaning of mechanical ventilation in three days. A new CT pulmonary angiogram was performed one day later, which excluded the presence of air in the entire pulmonary vascular tree and in the right heart chambers (Figure 5). After five days of being admitted to the ICU, she was transferred to the Intermediate Care Unit. At hospital discharge, 10 days after the event, she had no neurological sequelae and no need for supplemental oxygen.

## Discussion

Venous gas embolism occurs in 11%-23% of patients undergoing contrast-enhanced CT, and it is described that even a subclinical gas embolism can be detected immediately after intravenous injection of contrast medium. Contrast can be administered via an injector or manually, showing an incidence of gas embolism of 11.7% and 23%, respectively [1]. However, the frequency of air embolism and the injection rate, the injection site, or the amount or type of contrast agent have not been associated. Thus, careful handling of contrast material injections, attention to tube connections, and careful priming of the syringe and tubing used for the injection can reduce the risk of administering air. Inadvertent injection of air may be more common with automatic injection because there is no human who is directly looking at the syringe when it is injected into the patient [2]. Furthermore, contrast infusion via the injector must be interrupted before the reservoir is completely empty [1]. On the other hand, the incidence of passive venous air embolism during manipulation, insertion, or removal of CVC has been reported to be 0.13%, with most cases occurring during insertion of a tunneled catheter through a peel-away sheath. Careful CVC manipulation is crucial, particularly during the administration of therapy followed by the closure of its lumen [3].

Although air emboli can occur anywhere in the venous system, some locations are more common. The most common locations are the main pulmonary artery (60%), left brachiocephalic vein (15%), right atrial appendage (20%), and superior vena cava (5%) [3]. The predilection for these locations is mainly attributed to the buoyancy of the air in a liquid medium that makes the bubbles rise toward the nondependent areas within the circulatory system [1]. They are also significantly determined by the positioning of the patient. Hence the use of repositioning maneuvers as part of the treatment for a venous air embolism.

The morbidity of venous gas embolism depends on the rate, volume and type of gas that is embolised. Air emboli become significant when the rapid and continuous introduction of air forms a collection of air (e.g., in the superior vena cava) that suddenly travels to the heart, forming a mass of blood, fibrin, and air that mechanically obstructs the right ventricular outflow tract. Alternatively, air and fibrin can enter the pulmonary capillary network, leading to the development of pulmonary edema through the release of histamine and serotonin, which results in pulmonary vasoconstriction and increased vascular permeability [2]. A fatal gas volume in a venous gas embolism is based on case reports and estimated to be 200-300 mL or 3-5 mL/kg; however, fatal cases with volumes as low as 100 mL have been described, in cases of communication between the venous and systemic circulations (e.g., patent foramen ovale, arteriovenous malformation, etc.). Another problem is the presence of an air blood interface which may activate the blood clotting mechanisms and lead to intravascular blood clot formation [1].

Clinical manifestations are nonspecific and may include skin pallor, diminished or absent peripheral pulses, chest pain, dyspnea, nausea and vomiting, altered consciousness, focal neurological deficits, seizures, vertigo, and amaurosis [1]. On physical examination, findings may include hypotension and “mill wheel murmur” on chest auscultation, which is an auscultatory sound caused by gas agitation in the cardiac chambers, being the only specific sign attributed to venous gas embolism. Ultrasound signs of increased pressure in the right heart chambers may also be present [1].

In the treatment of venous gas embolism, 100% oxygen must be started immediately to make the diffusion of nitrogen easier, thus decreasing the volume of the embolus. The patient’s positioning is also important, and the patient should be placed in the left lateral decubitus position and in the Trendelenburg position (Durant's position) to relieve the obstruction of the right ventricular outflow tract, facilitating the displacement of air to the apex of the right ventricle [2].

In cases where the patient is unconscious, chest compressions can help relieve the obstruction of pulmonary flow by forcing the air into the small pulmonary vessels. Palmon et al. reported that up to 50% of the right atrial air could be aspirated through a multihole catheter with the tip placed 2 cm below the cavoatrial junction [1]. Despite not being the first-line treatment in cases of venous gas embolism, hyperbaric oxygen therapy should be implemented if there is a risk of paradoxical embolism. It can be a useful treatment in severe cases, even if treatment is delayed from the onset of the condition. In a pressurized chamber, hyperbaric oxygen therapy exposes patients to 100% oxygen at atmospheric pressure two to three times greater than the pressure at sea level (>2000 mmHg). The objective is to reduce the size of the gas emboli, thus making gas reabsorption easier and improving tissue perfusion [2].

## Conclusions

Venous gas embolism is currently a more recognized entity due to the increased use of CT as a complementary diagnostic method. In this case, contrast injection could be a probable cause; however, the possibility of catheter manipulation as a starting point for venous gas embolization cannot be excluded. Early diagnosis and treatment are essential to improve the outcome of these patients, and a complete clinical history and alertness to the clinical setting in which venous gas embolism occurs are vital. Although most cases of venous air embolism are asymptomatic, physicians need to be aware of the hemodynamic consequences that may underlie severe cases and the correct approach to treat this event.
